# Transcriptional and protein structural characterization of homogentisate phytyltransferase genes in barley, wheat, and oat

**DOI:** 10.1186/s12870-023-04535-x

**Published:** 2023-10-31

**Authors:** Zhanghui Zeng, Yong Jia, Xiaoping Huang, Zhehao Chen, Taihe Xiang, Ning Han, Hongwu Bian, Chengdao Li

**Affiliations:** 1 College of Life and Environmental Sciences, Hangzhou Normal University, Hangzhou, 311121 China; 2 Zhejiang Provincial Key Laboratory for Genetic Improvement and Quality Control of Medicinal Plants, Hangzhou, 311121 China; 3 Western Crops Genetic Alliance, Murdoch University, Murdoch, WA 6150 Australia; 4 State Agricultural Biotechnology Centre (SABC), College of Science, Health, Engineering and Education, Murdoch University, Murdoch, WA 6150 Australia; 5 Institute of Genetic and Regenerative Biology, Key Laboratory for Cell and Gene Engineering of Zhejiang Province, College of Life Sciences, Zhejiang University, Hangzhou, 310058 China; 6 Department of Primary Industry and Regional Development, Government of Western Australia, South Perth, WA 6155 Australia

**Keywords:** Antioxidants, Evolution, Homogentisate phytyltransferase, Protein structure modelling, Substrate docking, Transcriptome, Tocopherols, Vitamin E

## Abstract

**Background:**

Homogentisate phytyltransferase (HPT) is the critical enzyme for the biosynthesis of tocopherols (vitamin E), which are the major lipid-soluble antioxidants and help plants adapt to various stress conditions. *HPT* is generally strictly conserved in various plant genomes; however, a divergent lineage *HPT2* was identified recently in some *Triticeae* species. The molecular function and transcriptional profiles of *HPT2* remain to be characterized.

**Results:**

In this study, we performed comprehensive transcriptome data mining of *HPT1* and *HPT2* in different tissues and stages of barley (*Hordeum vulgare*), wheat (*Triticum aestivum*), and oat (*Avena sativa*), followed by qRT-PCR experiments on *HPT1* and *HPT2* in different tissues of barley and wheat. We found that the common *HPT1* genes (*HvHPT1*, *TaHPT1*s, and *AsHPT1*s) displayed a conserved transcriptional pattern in the three target species and were universally transcribed in various tissues, with a notable preference in leaf. In contrast, *HPT2* genes (*HvHPT2*, *TaHPT2*, and *AsHPT2*) were specifically transcribed in spike (developmentally up-regulated) and shoot apex tissues, displaying a divergent tissue-specific pattern. Cis-regulatory elements prediction in the promoter region identified common factors related to light-, plant hormone-, low temperature-, drought- and defense- responses in both *HPT1*s and *HPT2*s. We observed the transcriptional up-regulation of *HvHPT1* and *HvHPT2* under various stress conditions, supporting their conserved function in environmental adaption. We detected a clear, relaxed selection pressure in the *HPT2* lineage, consistent with the predicted evolution pattern following gene duplication. Protein structural modelling and substrate docking analyses identified putative catalytic amino acid residues for HvHPT1 and HvHPT2, which are strictly conserved and consistent with their function in vitamin E biosynthesis.

**Conclusions:**

We confirmed the presence of two lineages of *HPT* in *Triticeae* and *Aveninae*, including hexaploid oat, and characterized their transcriptional profiles based on transcriptome and qRT-PCR data. *HPT1s* were ubiquitously transcribed in various tissues, whilst *HPT2s* were highly expressed in specific stages and tissue. The active transcription of *HPT2s*, together with its conserved cis-elements and protein structural features, support *HPT2*s’ role in tocopherol production in *Triticeae*. This study is the first protein structural analysis on the membrane-bound plant HPTs and provides valuable insights into its catalytic mechanism.

**Supplementary Information:**

The online version contains supplementary material available at 10.1186/s12870-023-04535-x.

## Background

Tocopherols, together with tocotrienols, are the major forms of vitamin E, which are produced exclusively by photosynthetic organisms, such as plants, cyanobacteria, and algae [1]. Vitamin E is the major lipid-soluble antioxidant synthesized in plants and essential human diet nutrients [2, 3]. Despite its crucial nutritional value, studies show that the recommended dietary amount of vitamin E (15–30 mg/day) was often not met [4]. There are four isomers of tocopherols (α, β, γ, and δ), which differ only in the number and positions of methyl substituents on the chromanol ring [5]. Of these, α-tocopherol has the highest vitamin E activity, protecting polyunsaturated fatty acids from lipid peroxidation by quenching free radicals in cell membranes and other lipophilic environment, thus enhancing plant tolerance to biotic and abiotic stresses, such as cold, drought, salinity, heavy metal, high light, and bacterial infection [5–13].

The biosynthesis of tocopherols starts with the formation of homogentisate (HGA) via the shikimate pathway. Homogentisate phytyltransferase (HPT), together with homogentisate geranylgeranyl transferase (HGGT) are the key enzymes catalyzing the condensation of HGA and the saturated C20 isoprenoid phytyl diphosphate (PDP) and geranylgeranyl disphosphate to form 2-methyl-6-phytylbenzoquinone [14, 15]. Next, different isomers tocopherols were synthesized via methylations and cyclization of the aromatic ring, which are sequentially catalyzed by 2-methyl-6-phytylbenzoquinone methyltransferase, tocopherol cyclase, and γ-tocopherol methyltransferase [16–18]. Among the candidate genes in the tocopherol biosynthesis pathway, the biological function of *HPT* has been well elucidated in plants. In *Arabidopsis*, *HPT* is highly expressed in almost all of tissues and organs, including the root, leaf, flower and the embryo of caryopsis [18]. Overexpression of *Arabidopsis HPT* (*VTE2_1*) resulted in up to a 4.4-fold increase in total tocopherol levels in leaves and up to a 2-fold increase in seeds [19, 20]. On the other hand, the targeted mutation of *VTE2_1* completely eliminated tocopherol production, indicating this gene is the gene limiting tocopherol biosynthesis in *Arabidopsis* [21]. Tocopherol deficiency in *vte2* mutant resulted in severe defects in seed longevity, germination and early seedling growth [21] and increased sensitivity to low temperatures [12, 22]. In addition to *Arabidopsis*, the biological function of *HPT* genes in tocopherol production has also been confirmed in major crops such as barley [23], rice [24], oat [25], apple [26], and oil palm [27].

Despite the universal conservation of *HPT* in plants, the number of paralogous *HPT* genes can vary greatly across different species. In most cases, this is mainly caused by variations in their ploidy levels. For example, a single copy of *HPT* has been found in *Arabidopsis* [21], rice [28], tomato [29], oil palm [30], whilst 3 *HPTs* were reported in hexaploidy oat [25], 3 *HPTs* in hexaploid wheat [31], and 2 *HPTs* in rapeseed [32]. These observations indicate that the copy number of *HPT* tends to be highly conserved. However, our recent studies [23, 31] showed that a divergent lineage of *HPT2* is present in *Triticeae* plants such as barley and wheat, representing an interesting observation for this gene family. Particularly, the retention of the divergent *HPT2* in barley and wheat were suggested to be related to environmental adaption, such as cold tolerance [31]. In previous studies, the transcription pattern of *HPT* has been directly associated tocopherol accumulation in various crops [23, 26, 30, 33], which may play a critical role in plant growth under various stress conditions. Indeed, up-regulation of genes related to vitamin E biosynthesis and vitamin E accumulation has been reported in several species under low temperature [22, 34–37], light intensity [28, 38], drought [39], and bacterial infection [10]. Therefore, it would be interesting to investigate the transcriptional and molecular profiles of these divergent *HPTs* in *Triticeae*, which may have important implications for understanding their biological function and potential use in crop breeding for improved vitamin E content and stress tolerance.

The present study aims to characterize the transcriptional and protein structural profile of *HPT* in barley, bread wheat, and oat, and explore their impact on vitamin E synthesis. We searched *HPT* genes in 7 cereal crops and confirmed the presence of the divergence *HPT2* lineage in *Trticeae* and *Aveninae*. Transcriptional analyses based on qRT-PCR and public RNAseq data were performed for *HPT1* and *HPT2* in barley, bread wheat, and oat. Natural selection analyses identified potential amino acid sites under positive selection. Comprehensive protein structural modelling and substrate-docking analyses were performed to investigate the active sites responsible for tocopherol production. The potential application of this divergent *HPT2* lineage in crop breeding for enriched vitamin E content was discussed.

## Results

### The presence of a divergent lineage of homogentisate phytyltransferase (*HPT2*) in *Triticeae* and *Avenidae*

**Fig. 1:**
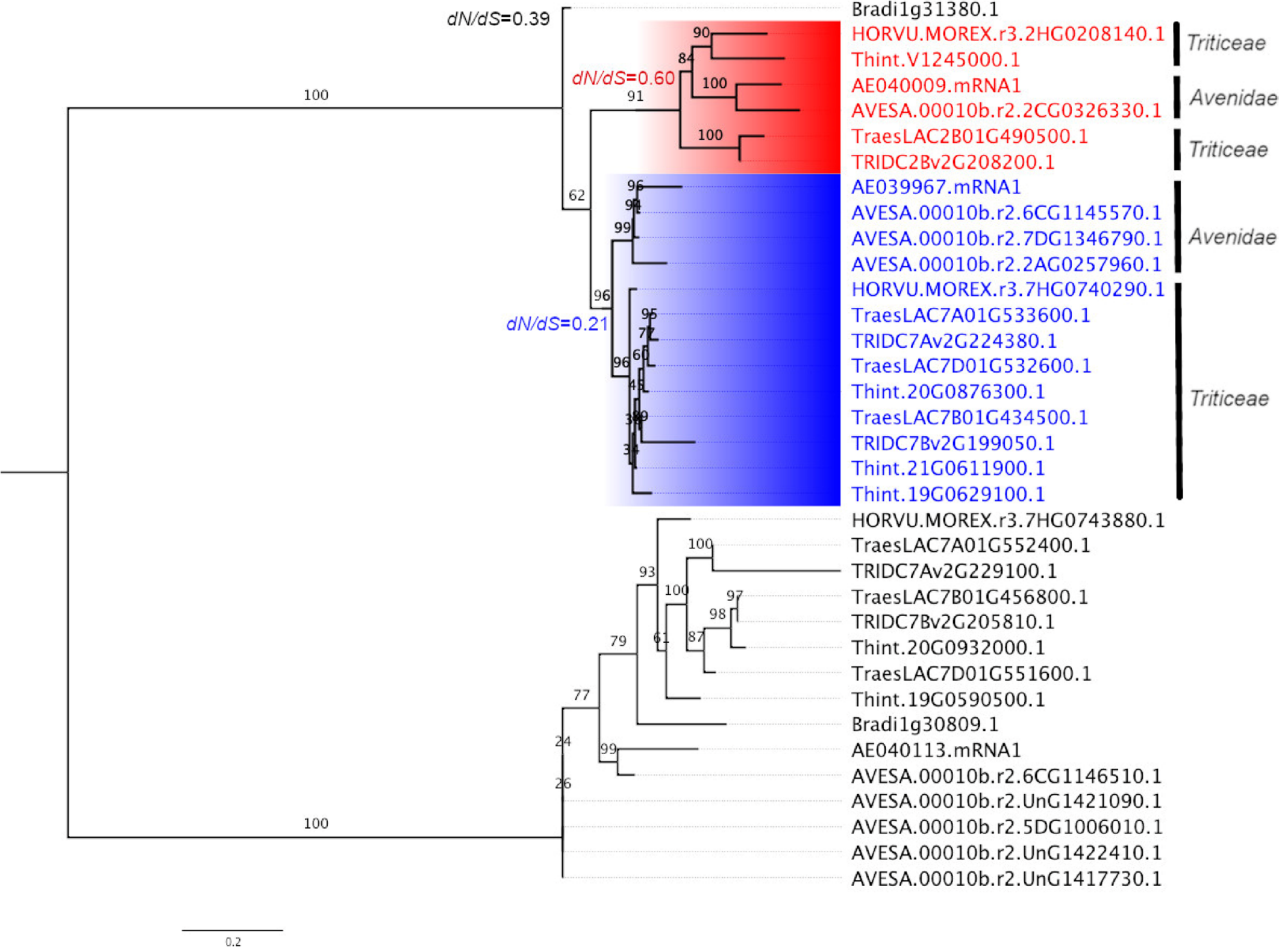
Displays the phylogeny of HPT and HGGT homologues in *Triticeae* and *Avenidae*. The phylogenetic tree was developed using ML method based on the amino acid sequence alignment. Branch supports based on 1000x bootstrapping were indicated above each branch. *HPT1* and *HPT2* lineages in *Triticeae* and *Aveninae* were highlighted in blue and red, respectively. Sequence alignment and phylogenetic tree are available at Supplementary Data S1. Natural selection pressure (d_N_/d_S_) for different lineages were indicated accordingly

Using HvHPT1 as the query sequence, we performed genome-wide screening of *HPT* homologues in 4 *Triticeae* (*H. vulgare*, *T. aestivum*, *T. turgidum* subsp. *dicoccoides*, *T. intermedium*), 2 *Avenidae* (*A. eriantha*, *A. sativa*), plus a reference species *B. distachayon*. The total gene number ranged from 2 in *B. distachayon*, 3 in *H. vulgare* and *A. eriantha*, 6 in *T. intermedium*, 7 in *T. aestivum*, to 9 in *A. sativa*. A maximum likelihood phylogeny was developed to examine their evolutionary pattern. As shown in Fig. 1, the target genes were divided into two major clusters, representing the *HPT* and *HGGT* lineages, respectively. In the *HGGT* lineage, the numbers of *HGGT* genes in the target species were generally consistent with their ploidy levels with the exception of *A. sativa*, which displayed a species-expansion with 5 *HGGT* genes, whilst *T. turgidum* has one only. In the *HPT* lineage, the single *HPT* gene in *B. distachayon* diverged first in accordance with the species phylogeny. Then, the *HPT* genes in *Triticeae* and *Avenidae* species diverged into 2 lineages, corresponding to a duplication event in their common ancestor as indicated in our previous study [31]. The first lineage *HPT1* (highlighted in blue) displayed a relatively shorter phylogeny branch and corresponds to the ancestral *HPT* gene in these species. The second lineage *HPT2* (highlighted in red) had a relatively longer phylogeny branch, indicating a more divergent sequence profile. In term of gene copy number, *HPT2* is preserved as a single copy in the 6 target species, while the number of *HPT1* is generally in agreement with species ploidy levels, with the exception of *T. intermedium* (tetraploid), which has 3 *HPT1* (Fig. 1). Noteworthy, *Triticeae* and *Aveninae* HPTs separated into 2 distinct clusters in the *HPT1* lineage, whilst barley HvHPT2 (HORVU.MOREX.r3.2HG0208140.1) and *T. intermedium* TiHPT2 (Thint.V1245000.1) seem to have a relatively closer relationship (Fig. 1; branch support 0.84) with *A. eriantha* AeHPT2 (AE040009.mRNA1) and *A. sativa* AsHPT2 (AVESA.00010b.r2.2CG0326330.1), implying an unusual amino acid sequence profile.

### Transcriptome analyses of *HPT2* and *HPT1* in barley, wheat, and oat

**Fig. 2:**
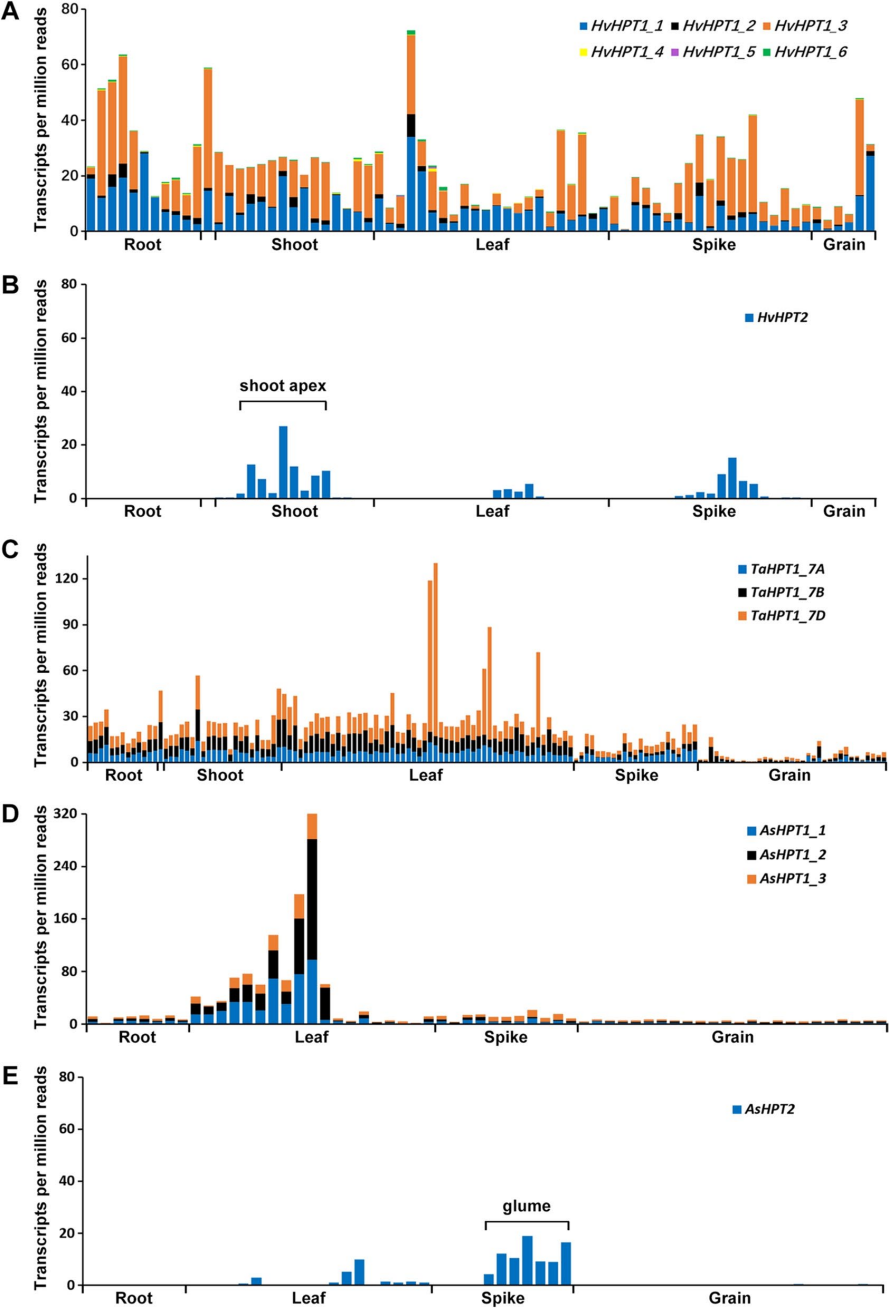
Transcriptional atlas of *HPT1* and *HPT2* in barley, wheat, and oat. **A** The transcriptional profile of *HvHPT1* (6 alternative transcripts). **B** The transcriptional profile of *HvHPT2*. **C** The transcriptional profile of *HPT1s* in bread wheat. **D** The transcriptional profile of *HPT1s* in oat. **E** The transcription profile of *AsHPT2* in oat. Transcriptome data for barley, wheat, and oat were obtained from EORNA [40], Wheat Expression Browser [41], and GrainGenes [42] databases, respectively. Corresponding transcriptional data was available in Supplementary Data S2

To explore the transcriptional profiles of *HPT1* and *HPT2*, public transcriptome databases in barley (https://ics.hutton.ac.uk/eorna/index.html), wheat (http://www.wheat-expression.com/), and oat (https://wheat.pw.usda.gov/GG3) covering various tissues and developmental stages were consulted. In barley, *HvHPT1* (6 alternative transcripts in Fig. 2A) was universally transcribed in all tissues, including root, shoot, leaf, spike, and grain. The highest expression of *HvHPT1* was observed in the leaf, followed by root and shoot, with relatively lower expression in grain. In contrast, *HvHPT2* (Fig. 2B) displayed a clear tissue-specific pattern and was only actively transcribed in the shoot apex and spike, and relatively weaker in the leaf. Furthermore, *HvHPT2* has a relatively weaker expression than *HvHPT1*. In bread wheat reference genome (Chinese Spring, EnsemblPlants release 56, https://plants.ensembl.org/Triticum_aestivum/Info/Index), where only *HPT1s* (*TaHPT1_7A*, *TaHPT1_7B*, *TaHPT1_7D*; Fig. 2C) were present, all three *HPT1s* were widely expressed in various tissues: the highest in leaf, followed by shoot, root, and spike, and the lowest in grain, which is similar to that observed for *HvHPT1*. Particularly, *TaHPT1_7D* seemed to be transcribed relatively higher than *TaHPT1_7A* and *TaHPT1_7B*, implying a potential sub-genome bias in gene transcription. In hexaploid oat, the three *HPT1* genes (*AsHPT1_1*, *AsHPT1_2*, *AsHPT1_3*; Fig. 2D) were also found widely transcribed in various tissues: exceptionally high in leaf, followed by root and spike, and the lowest in grain. In contrast, their *HPT2* homologue (*AsHPT2*; Fig. 2E) was specifically transcribed in the spike (glume) and weakly in the leaf. In summary, we observed a conserved transcription pattern for *HPT1* and *HPT2* across barley, wheat, and oat. Particularly, we found the divergent lineage *HPT2* was specifically transcribed in the spike (potentially in the husk or glume part), whilst *HPT1* was universally expressed in various tissues with leaf as the highest.

### qRT-PCR verification of the transcription of *HPT1* and *HPT2* in barley and wheat

**Fig. 3:**
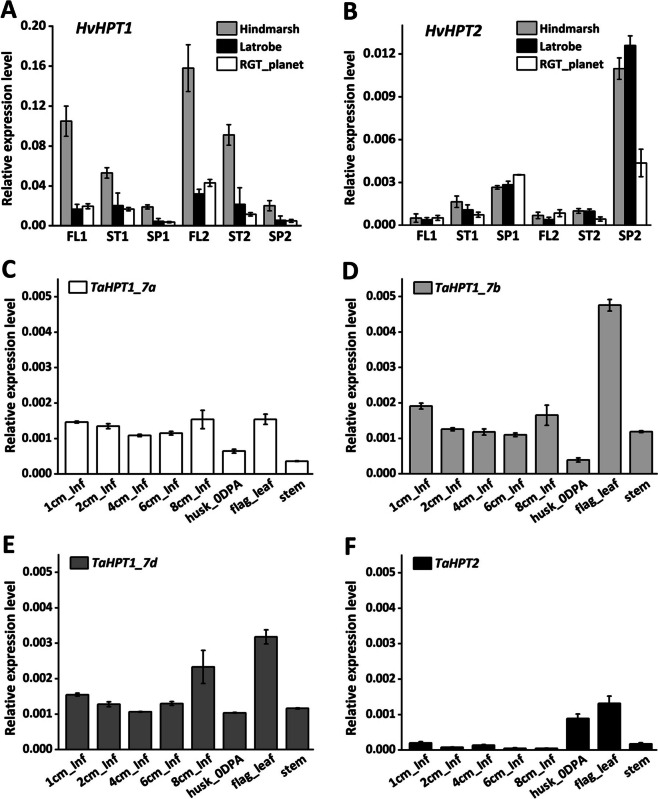
qRT-PCR analyses of *HPT1* and *HPT2* in barley and bread wheat. **A** *HvHPT1* and **B** *HvHPT2* in 3 tissues (FL: flag leaf; ST: stem; SP: spike) at 2 stages (S1: 3 cm inflorescence; S2: spike heading) in 3 cultivars. **C** *TaHPT1_7a*, **D** *TaHPT1_7b*, **E** *TaHPT1_7d*, and **F** *TaHPT2* in wheat cultivar Lancer (inf: indicate inflorescence; DPA: days post anthesis). Primer sequences are available at Supplementary Data S3

Due to tocopherols’ proven function under various stress conditions, the specific transcription of *HPT2* in spike tissue above is particularly interesting. To gain more insights and also verify the transcriptional profiles of *HPT1* and *HPT2*, qRT-PCR experiments were performed in barley and bread wheat lines containing the *HPT2* gene. In barley, three tissues (FL: flag leaf; ST: stem; SP: spike/inflorescence) at two stages (S1: 3 cm inflorescence; S2: spike heading) for three cultivars (Hindmarsh, Latrobe, RGT_planet) were analysed. Results (Fig. 3A) showed that *HvHPT1* was upregulated from stage S1 to S2 and was expressed the highest in FL, followed by ST, and least in SP. Among the three cultivars, Hindmarsh displayed consistently higher *HvHPT1* transcription, implying transcriptional variation across cultivars. In contrast to *HvHPT1*, *HvHPT2* (Fig. 3B) was preferentially transcribed in the spike tissue at both stages in all cultivars, consistent with public transcriptome data. Overall, *HvHPT2* seemed to display a relatively weaker transcription level than that of *HvHPT1*, consistent with the transcriptome data, although the qRT-PCR primer amplification efficiency needs to be calibrated to directly compare the expression levels of these two genes.

In wheat cv. Lancer, which contains the *HPT2* gene, inflorescence at five different stages (1 cm, 2 cm, 4 cm, 6 cm, and 8 cm) and flag leaf, stem, and husk (at the anthesis stage) was were studied. Overall, wheat *HPT1* genes (*TaHPT1_7a, 7b, 7d*; Fig. 3C and E) displayed conserved expression profiles across tissues and were all actively transcribed at comparable levels, the highest being in flag leaf, relatively lower in husk and stem. The preferential expression of wheat *HPT1* genes was similar to barley *HvHTP1* and was also consistent with the above transcriptome data. Compared to *HPT1s*, *TaHPT2* (Fig. 3F) also displayed relatively weaker transcription: the highest expression in flag leaf, followed by husk, but barely expressed in stem and inflorescences. The active transcription of *TaHPT2* in wheat husk and leaf is similar to that observed for *HvHPT2* and public transcriptome data.

### Cis-acting element, gene structure, and stress response analyses of *HPT1* and *HPT2*

**Fig. 4:**
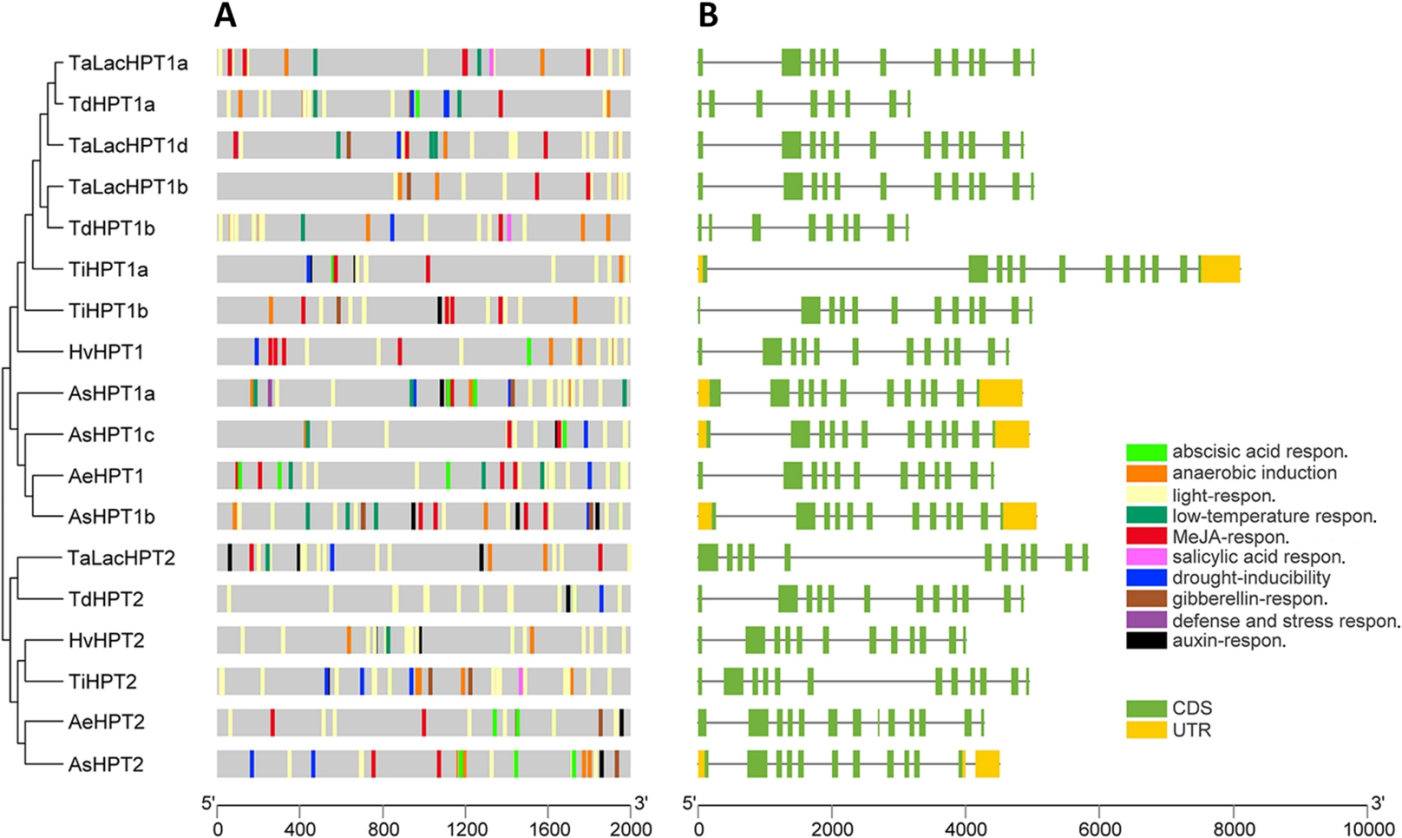
Cis-acting elements and gene structures of *HPTs* in 6 *Triticeae* and *Aveninae* species. The predicted cis-acting elements (**A**) and gene structure (**B**) were displayed using HPT phylogeny as the order. CDS: code region sequence; UTR: untranslated region. Corresponding promoter region sequences and cis-acting elementary prediction results are available in Supplementary Data S4

The transcriptional divergence between *HPT1* and *HPT2* indicated potential genetic variation in their regulatory regions. Therefore, in this study, we extracted the putative promoter regions of the *HPT1* and *HPT2* genes across the four *Triticeae* and two *Aveninae* species in this study and predicted the cis-acting elements. Results (Fig. 4A) showed that the promoter regions of both *HPT1* and *HPT2* were enriched with various cis-elements related environment adaption, particularly high in light-responsive and anaerobic induction, followed by low-temperature responsive and drought-inducibility, which is consistent with vitamin E’s well-known function in various stress tolerance. In addition, various cis-elements related to plant hormones, including abscisic acid, gibberellin acid, jasmonic acid, auxin, and salicylic acid were also identified. Noteworthy, for both *HPT1* and *HPT2* lineages, we noticed that abscisic acid elements were more prevalent in *Aveninae* than in *Triticeae*. Within the *HPT2* lineage, low-temperature element was only detected in *HvHPT2* and *TaHPT2*, whilst it is more enriched in most *HPT1* genes, indicating varied regulatory binding sites for these two lineages. Overall, we found generally conserved cis-elements in the putative promoter region of *HPT2*s, such as light responsiveness, anaerobic induction, drought-inducibility, and jasmonic acid responsiveness, supporting their potential function in stress adaption. Regarding gene structure, results (Fig. 4B) showed that *HPT1* and *HPT2* contain 12 exons with conserved exon length, supporting their close relationship as gene duplicates. The exceptions are *TdHPT1a* and *TdHPT1b* from *T. turgidum* subsp. *Dicoccoides*, which contained partial gene fragments compared to other *HPT*s, may be caused by gene annotation error.

To verify the transcriptional responses of *HPT1* and *HPT2* under stress conditions, the public barley transcriptome data with various stress treatments was consulted. Results (Supplementary Data S2, HvHPT_stress) showed that *HvHPT1* was ubiquitously transcribed in most barley tissues and were generally up-regulated under various stresses, such as disease infection (spot blotch), cold stress, waterlogging, drought, salinity, heat, and continuous light regimen. Consistent with our above transcriptional analyses, significant expression of *HvHPT2* was only observed in inflorescence and spike tissues of those varieties where it is present, such as Bowman (Supplementary Data S2). Notably, the transcription of *HvHPT2* could reach a comparable level with that of *HvHPT1* at specific tissues and stages, such as the shoot apex, awn primordium (5 mm), white anther (10 mm), apical, and inflorescence tissues (Project ID: PRJEB39672, PRJEB34648, and PRJEB8748; Supplementary Data S5), further supporting its critical role in inflorescence development. Interestingly, we also observed clear up-regulation of *HvHPT2* in the spike and inflorescence tissues under stress conditions, including long day (PRJEB8748), and drought (PRJEB12540), which are consistent with the cis-element prediction and support *HvHPT2*’s positive role in stress adaption.

### Natural selection analyses

To assess the evolution rates of *HPT1* and *HPT2* in *Triticeae* and *Aveninae*, we calculated the ratio (ω) of non-synonymous (*d*_*N*_) to synonymous (*d*_*S*_) substitutions using the branch-specific models based on the developed ML phylogeny (Fig. 1) in this study. Three lineages were specified for ω calculations: *HPT1*, *HPT2*, and background *HPTs*, corresponding to ω_HPT1_, ω_HPT2_, and ω_BG_, respectively. Under the three-ratio model (ω_*HPT2*_ ≠ ω_*HPT1*_ ≠ ω_*BG*_), ω_HPT1_, ω_HPT2_, and ω_BG_ were estimated at 0.20821, 0.60389, and 0.39162, respectively (Fig. 1; Supplementary Data S5), which showed that the selection pressure on *HPT2* is significantly higher than that from *HPT1*, indicating clearly relaxed selection pressure, whilst the latter is under relatively higher purifying selection.

### Protein structural modelling

**Fig. 5:**
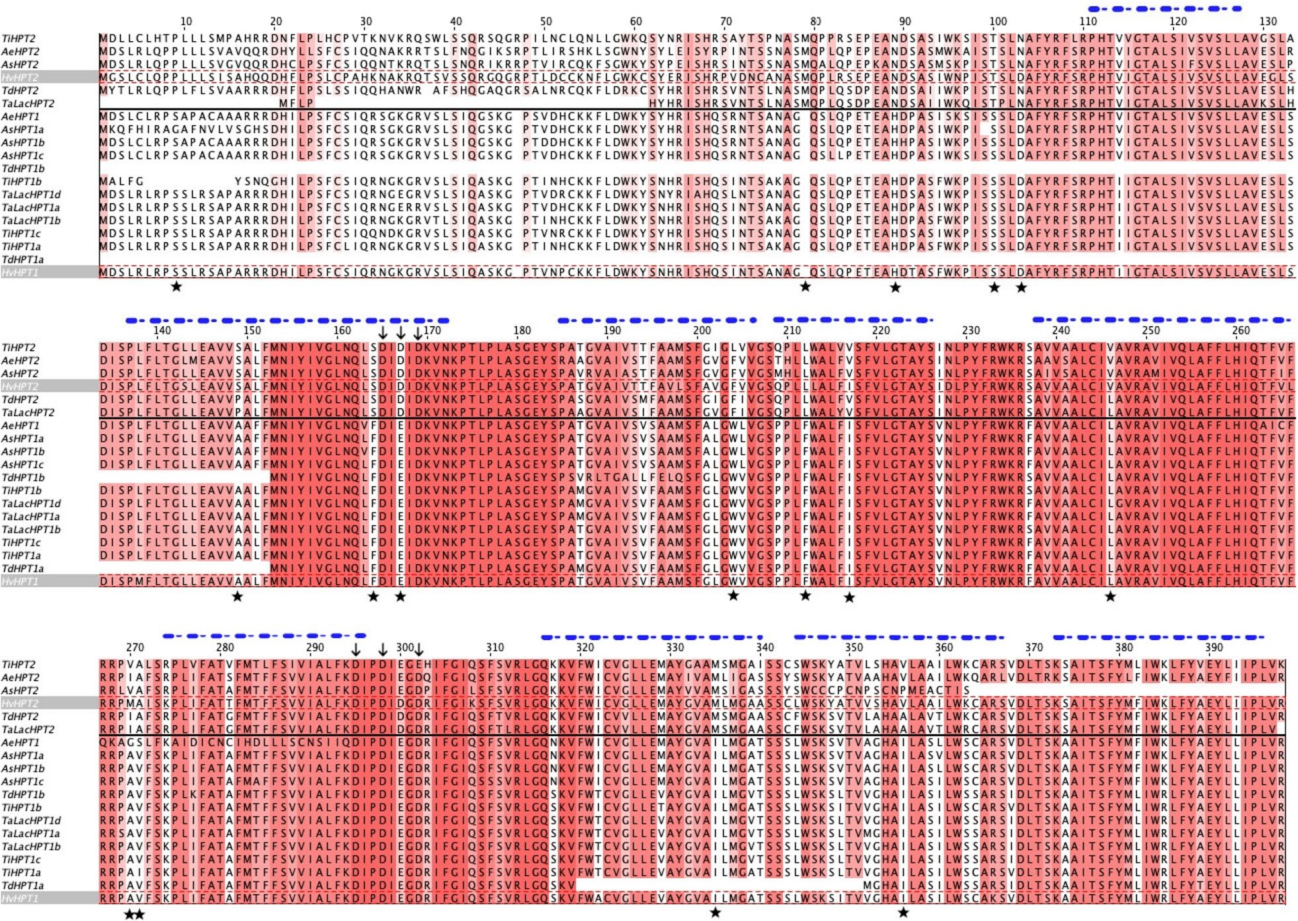
Amino acid sequence alignment of HPT homologues in *Triticeae* and *Avenidae*. Sequence alignment was trimmed and numbered using HvHPT2 as a reference. The red highlight indicates the conservation level at each site. HPT1s and HPT2s were separated by a black line. Amino acid sites under positive selection were underscored by a solid star symbol. Two conserved D-enriched motifs were indicated by downward arrows. Major helices were indicated by blue dashed lines above

To investigate if there were any potential functional divergence for HPT2 at the protein structural level, we performed amino acid sequence alignment for HPT1 and HPT2 in *Triticeae* and *Aveninae* (Fig. 5). Overall, HPT2s have 73.7% aa identity with HPT1s, whilst HPT2s and HPT1s share 80.7% and 90.7% identity with themselves, respectively, implying HPT2s are relatively more relaxed than HPT1s. The strong similarity between HPT2 and HPT1 implied that they may still share a potentially conserved function. Consistently, a highly conserved UbiA prenyltransferase family domain (aa125-aa386; PF01040) could be identified, which included two conserved aspartic-acid-enriched motifs DX[D/E]XD and DXXDXXXD (indicated by black arrows in Fig. 5), which are widely conserved in the UbiA superfamily [43]. Protein domain analyses indicated that both HvHPT1 and HvHPT2 are membrane-bound proteins, with an N-terminal cytoplasmic domain (aa 1-113 using HvHPT2 as reference), belonging to the HOMOGENTISATE SOLANESYLTRANSFERASE, CHLOROPLASTIC (PTHR43009) family. Among the 15 amino acid sites previously identified to be under positive selection in HPT2, most of them displayed clear amino acid substitutions in our sequence alignment (underscored by solid star symbols in Fig. 5).

**Fig. 6:**
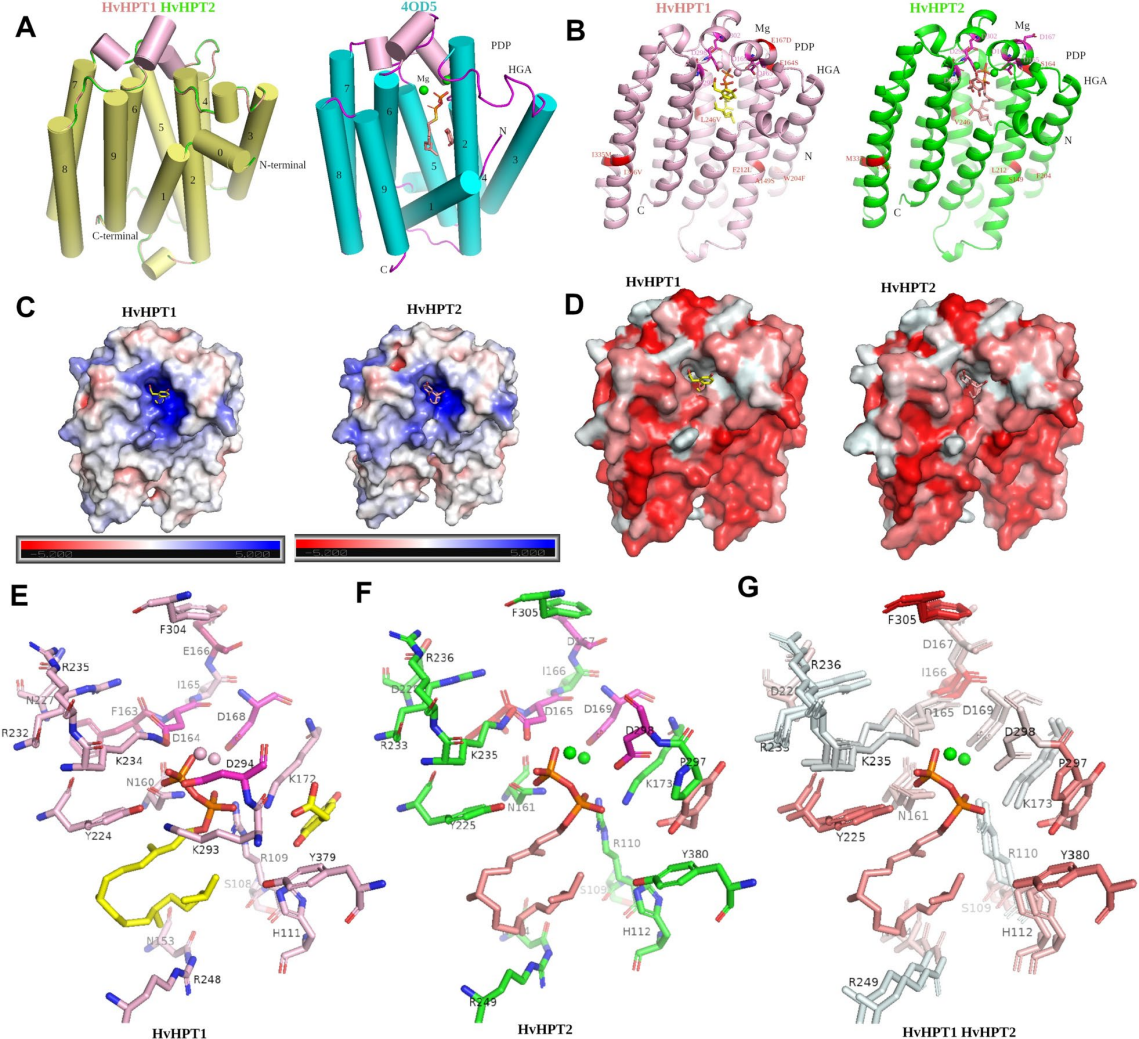
Protein structural modelling of HvHPT1 and HvHPT2. **A** Displays the overall structures of HvHPT1 and HvHPT2 (left) and 4OD5. **B** Displays the spatial positions of docked substrates Mg, HGA, and PDP, the interacting aspartic acid residues (pink), and positively selected amino acid sites (red) in HvHPT1 (left) and HvHPT2 (right). **C** Displays the electrostatic profiles of HvHPT1 and HvHPT2. **D** Displays the hydrophobicity profiles of HvHPT1 and HvHPT2. **E** Displays the substrate-interacting amino acids of HvHPT1 (numbering in HvHPT1). **F** Displays the substrate-interacting amino acids of HvHPT2 (numbering in HvHPT2). **G** Displays the hydrophobicity profile of the conserved substrate binding sites in HvHPT1 and HvHPT2 (numbering in HvHPT2). All amino acid site numberings were according to HvHPT2 unless otherwise specified. The generated protein models with docked substrate molecules are available in Supplementary Data S6

To assess the potential effect of these amino acid substitutions on enzyme activity, 3-dimensional structural models for HvHPT1 and HvHPT2 were created without the N-terminal cytoplasmic regions, which lack structural template. Substrate molecules homogentisate (HGA) and phytyl diphosphate (PDP) for HPT were also docked to the protein models to determine the substrate binding site. The overall folds of HvHPT1 and HvHPT2 are highly conserved, consisting of 10 main transmembrane helices (light orange in Fig. 6A-left; blue dash lines in Fig. 5) and connecting loops, similar to the archaeal UbiA protein (Fig. 6A-right; PDB: 4OD5) for which the protein structure has been experimentally determined [43, 44]. Based on structural superimposition with 4OD5, the substrate binding sites were determined to be located at the central cavity between the helix bundles. Notably, three short helices (Fig. 6A; highlighted in light pink) on top of the substrate binding pocket which cap the active sites after substrate binding [44] seem to be well conserved in our models. The two highly conserved aspartate-rich motifs (Fig. 6B; highlighted in pink) were located in these helix cap, coordinating the Mg^2+^ ion, playing an essential role in HPT function. The spatial location of the 15 selected amino acid sites were displayed (red) in Fig. 6B. Most of these residues are located on the transmembrane helices exposed to the exterior surface, except for 164S and 167D, which are positioned next to the conserved DXXXD motif (Figs. 5 and 6B). The electrostatic and hydrophobicity profile of HPT models were displayed in Fig. 6C and D, which showed that HvHPT1 and HvHPT2 have a conserved positively charged (thus hydrophilic) substrate binding pocket, which become more hydrophobic as it extends deeper into the transmembrane helices (Fig. 6C and D). Substrate docking in HvHPT1 and HvHPT2 indicated that the phytyl donor PDP may first bind deep into the active site, followed by HGA binding at the entrance.

Detailed examination of the interacting amino acid residues with the docked substrates revealed putative amino acid sites responsible for substrate binding in barley HPT. As shown in Fig. 6E (HvHPT1) and F (HvHPT2), 20 amino acid residues (109S, 110R, 112H, 154N, 161N, 165D, 166I, 167D, 169D, 173K, 225Y, 228D, 233R, 235K, 236R, 249R, 297P, 298D, 305F, 380Y) within 3 Å of the docked substrates were identified with putative substrate binding interactions, all of which were found be conserved between HvHPT1 and HvHPT2. The hydrophobicity profiles of these putative substrate binding residues are shown in Fig. 6G, where two modelled Mg^2+^ ions were coordinated by 165D and 169D, while 110R, 161N, 173K, 225Y, and 235K may be critical for the positioning of the di-phosphate groups in PDP, facilitating the cleavage and transfer of the phytyl group to HGA. The highly conserved substrate binding site for HvHPT1 and HvHPT2 supports the conserved function of HvHPT2 in tocopherol biosynthesis. In addition to those highly-conserved substrate-binding residues, amino acid substitutions between HvHPT1 and HvHPT2 were generally observed away from the substrate-binding sites, which may have little effect on substrate-binding. Indeed, under the optimal binding formations in our substrate docking analyses, the binding affinities for HGA and PDP to HvHPT1 and HvHPT2 were estimated at -10.72 and − 10.71 kcal/mol, respectively, similar to each other.

## Discussion

HPT is the crucial enzyme responsible for tocopherols biosynthesis in plants. Previous studies have shown that this gene is strictly conserved in various plant genomes [21, 28–30]. Despite varied copy numbers of HPT have been reported in some species, most of which are caused by different ploidy levels [25, 31], whilst species-specific duplication and divergent evolution of *HPT* have rarely been observed. However, in our recent studies [23, 31], we noticed a divergent lineage of *HPT2* present in *Triticeae* and *Avenieadeae*, which resulted from a dispersed gene duplication in their common ancestor. Most importantly, *HPT2* is only partially retained in some pangenome lines, which seems to be closely related to environmental adaption [31], implying an active role for *HPT2* in these plants. In this study, we confirmed the presence of *HPT2* lineage and found that *HPT2* is also present in hexaploid *A. sativa* cv. Sang, which has not been found before. The failure to identify *HPT2* in hexaploidy *A. sativa* was due to the use of *A. sativa* cv. OT3098 (V2), in which *HPT2* was not annotated. This further highlighted the limitation of using a single reference genome in genomic research.

The presence of a divergent lineage of *HPT2* in *Triticeae* and *Aveninae* motivated us to investigate its biological function. At the transcriptional level, *HvHPT2* was previously shown to be transcribed specifically in the husk tissue of barley grain [31]. In this study, we extracted public transcriptome data covering various tissues and stages and found that *HvHPT2* is not only transcribed in spike but also in shoot apex, suggesting additional biological functions for this gene. In addition, *TaHPT2* and *AsHPT2* were also found to be specifically expressed in the spike/glume, displaying a conserved expression pattern with *HvHPT2*. Considering that the major function of vitamin E in low temperature and other stress responses [45], the specific transcription of *HPT2s* in spike in this study is particularly interesting, indicating that *HPT2* may play a particular role in protecting these vulnerable tissues under stress conditions. Indeed, we found various plant hormones (auxin, GA, JA) responsive cis-regulatory elements in the promoter regions of *HPT2*s. Low-temperature responsive cis-regulatory elements were also detected for both *HvHPT2* and *TaHPT2*, albeit not for the other *HPT2s*. In barley, we confirmed the preferential transcription of *HvHPT2* in the young developing spike tissues using qRT-PCR in three Australian barley cultivars, which also showed clear transcriptional variations of *HvHPT2* across different cultivars. Future studies may be needed to identify high-expression alleles for *HvHPT2*, which may be exploited for breeding and genetic engineering purpose. Particular attention should be given to those wild barley lines collected from Tibet, in which *HvHPT2* was found to be generally conserved [31]. The absence of *HPT2* in the bread wheat reference genome Chinese Spring limited our ability to uncover its complete expression profile in different tissues. However, qRT-PCR in one wheat cultivar, Lancer containing *TaHPT2*, showed that *TaHPT2* was preferentially transcribed in the husk and flag leaf tissues, implying both shared and divergent expression profiles with *HvHPT2*. Further study may investigate its transcription in other wheat lines containing *HPT2*. In addition to *HPT2s*, we also examined the transcriptional profiles of *HPT1s* in barley and wheat, which displayed a conserved and wide-spread expression pattern, consistent with those reported in other species and studies [18, 25, 27, 28, 46].

Due to HPTs’ functioning as membrane-bound proteins, the experimentally determined structure of proteins in the UbiA superfamily is very limited, which hindered our understanding of the enzyme function of HPT at the protein structural level. To date, only two membrane-bound prenyltransferases from archaeal organisms have been structurally characterized [43, 44]. As far as the authors are concerned, our study is the first to create 3D models for plant HPTs and identified their putative substrate binding sites by substrate docking, revealing valuable structural insights into plant HPTs in tocopherol biosynthesis. Identifiying substrate-interacting amino acid residues may allow future engineering highly specific and efficient HPT mutants, which can be used for improved vitamin E production in crop breeding and genetic engineering. Indeed, transgenic expression of both HPT and HGGT has been widely performed to improve vitamin E content in many important crops such as cotton [47], barley [23], soybean [48, 49], tomato [26, 50], and lettuce [51]. The catalytic efficiency of HPTs from different species is clearly different. Our structural models confirmed the critical role of the conserved DXXDXXXD and DX[D/E]XD motifs in substrate binding in the UbiA superfamily. We found these two motifs form short helices on top of the catalytic cavity and are responsible for Mg^2+^ coordination, similar as that observed for their archaeal counterparts [43, 44]. Despite of only 73.7% amino acid identity between HPT1 and HPT2, we found that all of the putative substrate binding sites were strictly conserved between these two lineages of proteins, consistent with our previous transgenic expression analyses of HvHPT2, which was shown to be functional in tocopherol biosynthesis [31]. Moreover, our substrate docking analyses indicated that HvHPT1 and HvHPT2 may have similar substrate binding affinity, providing further support that HPT2 is a fully functional enzyme in tocopherol production.

Amino acid substitutions and transcriptional divergence are common observations during gene functional divergence following gene duplication [52]. Particularly, amino acid substitutions at the protein active sites could lead to immediate changes in either substrate binding specificity and/or binding affinity and play an important role in plant phenotypic diversification [53]. Well-known examples include the divergence between F3’H and F3’,5’H responsible for the red and blue anthocyanin productions, respectively [54], and the divergence between sorbitol dehydrogenase and L-idonic acid dehydrogenase for the sorbitol and tartaric acid productions, respectively [55]. Notably, within the vitamin E biosynthesis pathway, HPT and HGGT displayed varied substrate preferences [18], responsible for the production of tocopherols and tocotrienols, are also believed to be caused by amino acid substitutions in the active binding sites. Despite that we found no obvious substrate specificity changes between HPT1 and HPT2, the structural models created in this study may lay the foundation for future studies to uncover the molecular basis for the varied enzyme activities between HPT and HGGT. Many protein divergences after gene duplication lead to the accumulation of novel metabolites and confer environmental advantages are driven by natural selection [53, 55, 56]. We previously detected 15 amino acid sites in HPT2 under positive selection compared to HPT1 [31], which suggested that the emergence and retention of HPT2 in *Triticeae* and *Aveninae* may be associated with plants’ environmental adaption. In this study, we examined the spatial location of these amino acid sites and found that these amino acid changes may not directly affect substrate binding, but instead are likely related to membrane interaction due to their generally presence in the exterior surface of the protein structure. The biological significance of these selected amino acid changes in HPT2, together with its potential use in crop breeding and genetic engineering need to be examined with further in-depth functional analyses.

## Conclusions

We confirmed the presence of two lineages of *HPT* in *Triticeae* and *Aveninae*, including hexaploid oat and characterized their transcriptional profiles based on transcriptome and qRT-PCR data. We found that *HPT1s* were ubiquitously transcribed in various tissues and species, whilst *HPT2s* were only highly expressed in specific stages and parts of the spike tissue. We revealed the transcriptional up-regulation of *HvHPT1* and *HvHPT2* under various stress conditions in barley. The active transcription of *HPT2*, together with its conserved cis-elements and protein structural features, support *HPT2*s’ active role in tocopherol production in *Triticeae*. will facilitate future breeding for cereal crops with improved tocopherol content and nutritional values. This study is the first protein structural analysis on the membrane-bound plant HPTs and provides valuable insights into its catalytic mechanism.

## Methods

### Identification of HPT homologues and phylogeny development

Genomic datasets for barley (*H. vulgare* cv. Morex V3), wild emmer (*Triticum turgidum* subsp. *dicoccoides* Zavitan V2), bread wheat (*T. aestivum* cv. Lancer V2.1), *Thinopyrum intermedium* (V3.1), *Avena eriantha* (id53381), *A. sativa* cv. Sang (V1.1), and *Brachypodium distachyon* (V3.1) were downloaded from Phytozome 13 (https://phytozome-next.jgi.doe.gov/), GrainGenes (https://breadwheat.pw.usda.gov/GG3/), EnsemblPlants (release 56, http://plants.ensembl.org/info/index.html), or the species-specific website and were used for gene identification. Genuine HPT homologues were identified using the method as described in our previous study. Amino acid sequence alignment was performed using MUSCLE program (8 iterations) [57]. Phylogeny was developed using IQ-TREE program [58] with JTT + G4 substitution model. Branching support was calculated based on bootstrapping for 1000 times. Final tree annotation was performed in Figtree (v1.4.3, http://tree.bio.ed.ac.uk/software/figtree).

### RNA extraction and qRT-PCR analyses

Barley and wheat plants were grown in pots under a natural light glasshouse. Plant tissues were frozen in liquid nitrogen immediately after sampling and were ground into a fine powder using a pre-cooled pestle and a mortar. Total RNA was extracted using ∼100 mg of grounded powder using Trisure® (Bioline, Australia). SensiFAST™ cDNA Synthesis Kit (Bioline, Australia) was used for cDNA library construction following product instructions.

SensiFAST™ SYBR No-ROX Kit (Bioline, Australia) was used for RT-qPCR experiments with a reaction volume of 10 µl, containing 5 SensiFAST mix, 4.2 µl cDNA sample, 0.8 µl primers (500 nM). For each sample, three biological replicates with 2 technical replications were included. ViiA7 Real-Time PCR System (Thermo Fisher Scientific, United States) was used for the RT-qPCR reaction. RT-qPCR primers specificity was validated by melting curve analyses. Reference genes *HvActin* in barley and *TaACT-1* in wheat [59] were used. Gene expression values were calculated using the comparative Ct method (2^–ΔCt^).

### Transcriptome data mining

The transcriptional data for HPT-encoding genes were extracted from EORNA RNAseq database (https://ics.hutton.ac.uk/eorna/index.html) and BarleyExpDB (http://barleyexp.com/index.html) for barley [40, 60], Wheat Expression Browser (http://www.wheat-expression.com/) for bread wheat [41], and GrainGenes (https://wheat.pw.usda.gov/GG3/). Raw transcriptional value in transcripts per million read was plotted using Microsoft Excel software.

### Promoter binding motif and gene structure analyses

Cis-regulatory elements were predicted using PlantCARE (http://bioinformatics.psb.ugent.be/webtools/plantcare/html/) website. The 2000 bp sequences upstream of the translation start site for target *HPT* genes were used as input for PlantCARE prediction. Gene model information was extracted from each species’ corresponding genome annotation files. The identified cis-elements and gene structures were visualized using TBtools program [61] together with the developed phylogeny as a guide.

### Natural selection analyses

Natural selection pressure was assessed by measuring the ratio of non-synonymous to synonymous substitutions (ω = d_N_/d_S_). Codon-based maximum-likelihood estimates of ω was performed using codeml in PAML4.7 [62]. Codon-based alignment of conserved domain sequences was carried out using MUSCLE. Alignment was trimmed manually using *HvHPT2* as the reference. The sub-tree covering *HPT* only was used as input for codeml. Branch pattern specification was implemented using Treeview1.6.6 (http://taxonomy.zoology.gla.ac.uk/rod/treeview.html).

### Protein structural modelling and substrate docking

Protein structural modelling was performed using Google AlphaFold tool [63] using an online interface at https://colab.research.google.com/github/sokrypton/ColabFold/blob/main/AlphaFold2.ipynb. The amino acid sequences of HvHPT1 and HvHPT2 were used as input. For each protein, five models were generated and examined. Only the top-ranking model was selected for downstream analysis. Autodock Vina tool [64] was employed to dock small molecules to the selected model with docking parameters energy_range = 4 kcal/mol, and exhaustiveness = 8. MGLTools (https://ccsb.scripps.edu/mgltools/ ) was used for docking file preparation. For each receptor and ligand combination, the top 9 ranking conformations were evaluated. PyMol (Schrödinger, LLC. Version 2.4.0, http://www.pymol.org/pymol) was employed for model visualizations in this study.

### Supplementary Information

**Additional file 1: Supplementary Data S1.** Sequence alignment and phylogenetic tree files. **Supplementary Data S2.** Retrieved transcriptome data for the target HPT genes. **Supplementary Data S3.** Primer sequences used in qRT-PCR. **Supplementary Data S4.** Putative promoter region sequences and cis-acting element prediction results. **Supplementary Data S5.** Natural selection output files. **Supplementary Data S6.** Generated protein models of HvHPT1 and HvHPT2 with docked substrates HGA and PDP in the optional conformation.

## Data Availability

Supplementary files deposited at figshare (DOI: 10.6084/m9.figshare.22983752).

## Abbreviations

HPT: Homogentisate phytyltransferase
HGGT: Homogentisate geranylgeranyl transferase
PDP: Phytyl diphosphate
FL: Flag leaf
ST: Stem
SP: Spike/inflorescence
*d*_*N*_: Non-synonymous substitutions
*d*_*S*_: Synonymous substitutions
